# Wearable Embedded Intelligence for Detection of Falls Independently of on-Body Location

**DOI:** 10.3390/s19112426

**Published:** 2019-05-28

**Authors:** José Alves, Joana Silva, Eduardo Grifo, Carlos Resende, Inês Sousa

**Affiliations:** Associação Fraunhofer Portugal Research, Rua Alfredo Allen 455/461, 4200-135 Porto, Portugal; joana.silva@fraunhofer.pt (J.S.); eduardo.grifo@fraunhofer.pt (E.G.); carlos.resende@fraunhofer.pt (C.R.); ines.sousa@fraunhofer.pt (I.S.)

**Keywords:** automatic fall detection, wearable solutions, inertial sensors, accelerometer, state machine, sampling rate

## Abstract

Falls are one of the most common problems in the elderly population. Therefore, each year more solutions for automatic fall detection are emerging. This paper proposes a single accelerometer algorithm for wearable devices that works for three different body locations: chest, waist and pocket, without a calibration step being required. This algorithm is able to be fully executed on a wearable device and no external devices are necessary for data processing. Additionally, a study of the accelerometer sampling rate, that allows the algorithm to achieve a better performance, was performed. The algorithm was validated with a continuous dataset with daily living activities and 272 simulated falls. Considering the trade-off between sensitivity and the number of false alarms the most suitable sampling rate found was 50 Hz. The proposed algorithm was able to achieve a trade-off of no false alarms and 89.5% of fall detection rate when wearing the sensor on the user’s waist with a medium sensitivity level of the algorithm. In conclusion, this paper presents a reliable solution for automatic fall detection that can be adapted to different usages and conditions, since it can be used in different body locations and its sensitivity can be adapted to different subjects according to their physical activity level.

## 1. Introduction

Among the elderly population, falls are one of the most common causes of death and injury. More than 30% of people over 65 years old fall each year and the prevalence increases for people above 80 years old [1]. Even a minor fall can severely affect the physical and mental health of an elder due to the fear of falling again. Thus, the elderly quality of life and of their carers can be affected [2]. Besides the social and personal effects, falls play an important role in health-care costs. For instance, in 2015 the direct costs for fatal and non fatal fall injuries were of 637.5 million and 31.3 billion dollars respectively [3]. Some studies have made some relevant developments on fall prevention through gait stability assessment. Schooten et al. [4] performed a study using wearable sensors to analyze the relation between common gait characteristics and the time to the next fall. Their findings reveal that with the daily measurement of these gait characteristics it is possible to assess the elderly risk of falling. Although it can contribute to prevent falls, the occurrence of falls is not only dependent of the gait stability of the users but also from external perturbations as are for instance objects that the involving environment contains [5]. For this reason, the occurrence of falls is not always predictable. Therefore, it urges the need to be able to detect the falls at the moment they occur. An earlier detection of a fall will allow a faster intervention, decreasing the severity of injuries and, in some cases, avoiding deaths [6]. Therefore, in the past years, the scientific community is making an effort in order to develop systems for automatic fall detection [7].

Automatic fall detection systems, following [8], are divided in three main types: wearable device based, ambient device based and vision based. The main problem with ambient device and vision based systems is the restriction of its use to the room where the sensors/cameras are placed. Additionally, they usually require a complicated installation and setup when compared with wearable sensors based systems [8]. Smartphones’ embedded sensors have also been used for fall detection [9,10], however, in some situations they are not carried by the user or the in the user’s clothes, which will disable this specific function. Therefore, approaches based on wearable sensors, even though being more intrusive and less accurate [8], emerge as a potential solution to overcome these problems. As reported in [11] many proposals involving wearable sensors have been studied with the aim of solving the problem of automatic fall detection. The performance reported in such studies depends on several variables, for instance, the type of algorithm used, type of sensor, number of sensors, sensor position and type of data used for training.

One of the main problems when developing a fall detection algorithm is the data availability [12]. Falls rarely occur in a normal daily routine and for this reason acquisition of real fall data is really hard and time consuming. However, some research projects as the FARSEEING consortium have tried to aggregate signals from elderly falls from different places all over the Europe. Until 2015 only 209 valid falls were acquired. However, during this acquisitions inertial sensor data was collected in just two different positions: thigh and back (L5 vertebra) [13]. Therefore, the development of an algorithm using this data is restricted to a few amount of samples and to these specific positions. Techniques of data augmentation have been used with these real falls in some studies. However, these methodologies can introduce some bias in the results [12]. Therefore, most of the studies presented in literature have been using simulated falls data in order to train the algorithms. Some studies have reported significant differences of the simulated falls when compared with real falls data [14], however, some have found that some characteristics are common between both types of falls [15]. Others have used a mix between real fall data and simulated data, both simulated falls and ADL [16] in order to train and test a fall detection algorithm. It was verified that the best training method should be either to use the same type of data for training and testing, or the use of a mixed dataset of simulated and real data.

Most of the fall detection algorithms developed for wearable devices are based on simulated data acquired from a single [15,17,18,19] or multiple accelerometer sensors [20]. Often multiple inertial sensors are used, for instance, the combination of gyroscope and accelerometer [21,22] or accelerometer and barometer [23]. Regarding the type of algorithm, works vary from the most simple threshold-based algorithms [15,17,18,24] to machine learning algorithms [20,25]. As the number of sensors and algorithm complexity increases, more processing power will be required in order to execute the algorithm. This can be achieved with more powerful and expensive devices or by streaming the data to a more capable device, like a smartphone, to process the data [18]. However, in this case the need to always carry the smartphone is not eliminated [9,10]. Also, the use of various sensors can be uncomfortable for the subject. Urges then the need of a single body-worn unit with inertial sensors that can be carried everywhere and used in most daily situations [8].

The review by Schwickert et al. [11] concluded that there is no consensual position where the sensor should be placed in order to achieve the better fall detection rate. The most used positions are usually the waist [17,19,24], hip and trunk [18]. Some studies compared the performance of their algorithms for different positions. Kangas et al. [26] compared the performance of the algorithm in three body positions: wrist, waist and head, and concluded that the head and waist would be the most suitable positions. Furthermore, Gjoreski et al. [27] studied the best accelerometer placement for posture recognition and fall detection and concluded that both waist and chest positions had the best performance among waist, chest, thigh and ankle. However, similarly to the smartphone, sometimes the sensors cannot be placed in a specific position due to user’s health condition or acceptability. Pannurat et al. [18] have tackled this problem by developing an algorithm that works in several positions of the body such as the head, upper arm, wrist, ankle, chest, waist and thigh. However, this algorithm requires a calibration step for the position where the sensor will be used, and an external computer to perform the algorithm computation. If the person wants to change the position of the sensor, a new calibration step is always required. Since the user may not be an expert, the calibration step might be performed wrongly and this may hinder the proper functioning of the system.

This work focus on the development of a fall detection algorithm for a wearable device. The algorithm is designed to execute entirely on the wearable device, which has a local processor with limited battery, computational and memory capabilities. Thus, the developed algorithm incorporates multiple adaptations to accommodate the complexity and energy consumption restrictions of the wearable device. In order to maximize energy use efficiency, a single accelerometer sensor is used. A study of the optimal sampling rate to maximize the fall detection performance is also presented. Since the wearable device can be placed in different on-body positions, a generic fall detection algorithm has been developed, avoiding the need for calibration anytime the sensor position is changed. A general study on the best combination of sampling rate, sensitivity level and sensor position was also performed in order to optimize the algorithm. The main objective is to obtain a reliable fall detection algorithm that can be deployed in a wearable device with limited computational power. This algorithm should also work properly independently of the position where the wearable device is placed. The best positions found in literature, chest, waist and pants frontal pocket, were considered.

## 2. Materials and Methods

In this chapter the collection of datasets for training and validating the algorithm is described in Section 2.1, followed by the definition of the fall detection algorithm, Section 2.2, and its implementation in the wearable device, Section 2.3. Furthermore, a method to study the accelerometer sampling rate that best fits our algorithm is presented in Section 2.5 and the algorithm optimization process is presented in Section 2.6.

### 2.1. Datasets

In order to train the proposed fall detection algorithm, 3 axial accelerometer data from simulated falls and non-fall movements were collected, as presented in Table 1. This dataset will be referred in the remaining document as *DS-1*. Besides ambulatory movements, some data from movements that, due to their hard impacts, could more likely trigger false alarms (FAs) were also collected. One of the most common movements performed during daily usage is to lay the sensor on a table when the user will not use it, or, for instance, to charge the sensor. During a preliminary study it was verified that this type of movement was one of the most likely to trigger false positives (FPs). Therefore a high amount of movements of this type were acquired to train the algorithm. Another type of non-fall movements acquired were, for instance, getting up, bend and pick up an object from the floor, as shown in Table 1, based in procedure presented in [6,25]. The acquisitions were made using the sensor inside the user’s pants/shorts frontal pocket, on the waist (fixed on the belt) or on the chest. Data were collected from 19 subjects, 5 women and 14 men, with an average age of 25 ± 2 years old who gave their informed consent and participated voluntarily in the data acquisition. The simulated falls were performed in ambulatory conditions and the users fell to a 10 cm high gym mattress, while wearing a helmet for their safety. This dataset includes 1399 non-fall movements (6.5 h of data) and 1009 simulated falls (4.5 h) in a total of 2408 movements (11 h of data). Each sample acquired is considered as an activity, i.e., each sample acquired of walking a few meters is considered as a single activity that can be well classified, non-fall, or misclassified, fall.

To validate the algorithm in similar conditions to daily life, 22 young subjects, 5 women and 17 men, average age of 26 ± 3 years old, have performed a continuous data collection including non-fall movements intercalated with falls, referred as dataset *DS-2*. From these 22 young subjects only 9 subjects have participated in the data collection of the dataset *DS-1*. However, from those 9 subjects, 7 have only performed non fall movements. The remaining two have contributed with data from simulated falls and non fall movements. In this acquisition, each subject performed 6 min of each of the following ambulatory activities: standing still, sitting, walking, running, standing with freedom of movements and laying. Between these activities, each subject has performed 4 different simulated falls. The complete protocol had a duration of approximately 40 min per subject. Regarding the type of simulated falls, the subjects were divided in two different groups. A group of eleven subjects (1 woman, 10 men) have performed a forward fall, a backward fall, a sit-stand transfer fall and a stand-sit transfer fall. The remaining eleven subjects (4 women, 7 men) have performed movements of stumble and fall forward, lateral fall, vertical fall (faint simulation) and a forward fall preceded by getting up and walking for a few meters. These movements were chosen based in the procedures presented in [6,25]. During the acquisitions, each subject was wearing 3 wearable devices, in the three considered on-body positions, chest, waist and pocket (14 subjects used it in the right pocket and 8 used it in both pockets). Eight of these subjects were wearing an additional sensor, making a total of 4 devices, placed in the chest, waist and both left and right pants/shorts frontal pockets. A total of 44.67 h of accelerometer data containing 272 falls were acquired. The algorithm was tested individually for each subject and the overall performance was analyzed.

### 2.2. Fall Detection Algorithm

The proposed algorithm is a modified version of the state machine algorithm developed in our previous work [9] adapted to be implemented in a wearable device, Figure 2 from [9]. Although the structure of the state machine is the same, since the device can be placed more freely on the body, the conditions of transition between states are different, as well as the thresholds of each state. The features used in the smartphone algorithm were dependent of the orientation of the device. This new version of the algorithm should work independently for three different positions. In these positions the device will have different orientations. Therefore, as we do not want to require a calibration step the calculation of features was changed in order to make the features between each state independent of the wearable device orientation. The objective is for the user to wear the wearable in the most convenient place without having additional concerns. So, the thresholds between each state are defined by a feature that characterizes each new state. These features were defined in our previous study of Aguiar et al. [9] using machine leaning techniques in order to obtain the best features that characterize each phase of the fall. Then the best features obtained using these machine learning techniques were used in order to build the state machine. To transit from state to state, a single or multiple features are calculated from the accelerometer data. This data is processed in real time and sample by sample. If the value of the calculated feature crosses the threshold previously defined, the transition between consecutive states is performed. In this algorithm, in order to trigger a fall event, all the fall stages should be successively detected through the analysis of the data collected by the accelerometer placed on the user’s body. The state machine has five different states: Stable, Unstable, Falling, Impact and Unconscious Watcher, Figure 2 from Aguiar et al. [9]. The transitions between states follow:

1. **Stable to Unstable State**—When the fall detector algorithm is enabled and the subject is not moving, the system will start in the Stable state. Then, if some relevant acceleration changes are detected, the algorithm transits to the Unstable state. This change in acceleration is evaluated calculating the magnitude of the acceleration.
2. **Unstable to Falling State**—When in the unstable state, a significant decrease in acceleration can indicate that the user is experiencing a free fall, Falling State. To check if the decrease in acceleration values occur according to what is expected during a fall, the ratio between the magnitude of the linear acceleration and the magnitude of acceleration at each moment is evaluated.
3. **Falling to Impact State**—If a fall is truly occurring, when the subject hits the ground, a sudden and significant increase in the acceleration and a large difference in body orientation occurs, corresponding to the change from standing/sitting positions to the lying position occurring after the fall. Thus, when the state machine is in the falling state, two different features are evaluated: the magnitude of acceleration and the angle between two different vectors: the average acceleration vector in this state and the average acceleration vector obtained before entering on the Falling state.
4. **Impact to Broadcast of the fall**—After the impact, the system starts an Unconscious watcher that will check if the user has recovered from the fall or not. If the user does not move during five seconds after the fall, a fall alert will be broadcasted. If some movement is detected, the system will restart the process in the Unstable state. This detection of movement is accomplished by evaluating the values of the acceleration magnitude during the Unconscious watcher.

### 2.3. Wearable Implementation

The implementation of the algorithm presented in Section 2.2 on a wearable device is intrinsically dependent on the hardware that comprises it. Its processing and memory capabilities will impose a set of restrictions to the arithmetic operations used, the complexity of the algorithm and the amount of data that can be locally stored.

In this work the selected wearable device contains a ARM cortex M0+ processor, due to its low power and cost characteristics, allied with the set of basic resources needed to execute the fall detection algorithm. However, executing the algorithm on such a device demands a number of optimization steps to deal with the restrictions imposed by an M0+ processor, such as the unavailability of hardware divisions and floating point numbers. The computational and memory capabilities of this processor are insufficient to support the Java virtual machine needed to run the Java algorithm developed for a smartphone in our previous work [9]. Therefore the algorithm was converted to C.

Due to the nonexistence of hardware division or trigonometric calculations on the wearable Central Processing Unit (CPU), all the arithmetic expressions were rearranged in order to avoid such calculations, and optimize the computational complexity of the algorithm. The use of floating point numbers also requires considerable computational power, therefore, only fixed point numbers were used. The CMSIS library [28]—a library containing a set of software implementations of mathematical calculations optimized for processors with constrained processing resources—was then used to help manage the aforementioned operations.

In order to guarantee that the algorithm can run in the chosen wearable device we have performed some simulation tests of the algorithm running on a simulated CPU. In average the algorithm has spent 1.73 ms processing each sample of the dataset *DS-1*. Also, some memory tests were performed and the conclusion was that this algorithm requires just 350 bytes of memory RAM and 3 kB of ROM. Therefore, these simulations demonstrate that this algorithm can be deployed in this wearable device and run in real time.

### 2.4. Algorithm Evaluation

The fall detection algorithm developed does not have the objective of discriminating between each type of movement present in the datasets. The objective is to detect if a fall has or not occurred (binary classification). Therefore, the algorithm classifies every event either as a fall, positive event, or as a non-fall movement, negative event. Thus, in order to analyse the performance of this algorithm, it is necessary to evaluate the number of True Positives (TP), False negatives (FN), False positives (FP) and True negatives (TN) namely using metrics as the sensitivity (Sens), Equation (1), and specificity (Spec), Equation (2). However, in this work, the results are, in a first phase, analyzed by the Youden index or J-index, Equation (3), since its value is a combination between specificity and sensitivity. Also, as there is always an important trade-off between these two metrics, their particular values are also taken into account while evaluating and choosing the sets of thresholds.

(1) $$Sens=\frac{TP}{TP+FN}\times 100\%$$

(2) $$Spec=\frac{TN}{TN+FP}\times 100\%$$

(3) $$J_{index}=Specificity+Sensitivity-100\%$$

### 2.5. Accelerometer Sampling Rate Analysis

There is no consensus in the literature regarding the ideal accelerometer sampling frequency for fall detection. In the review of Schwickert et al. [11] 61 studies focused on the development of algorithms using accelerometer data for fall detection were analyzed and the range of sampling rates used varies from 6 to 3200 Hz. Furthermore, Kangas et al. [15] have used dynamic sampling frequencies depending on the fall phases. Therefore, it is important to study the sampling rate that would allow our algorithm to achieve the best performance. Still, the accelerometer used in our wearable device only allows sampling rates of 8, 50, 100, 250, 333 and 500 Hz. As the samples of the datasets *DS-1* and *DS-2* were collected at 100 Hz, the performance of the algorithm can only be tested with the data undersampled to 8, 50 or 100 Hz. In [15] the authors discussed that during the pre-impact phase of a fall, the data sampled at 6.25 Hz were not enough to identify the movements in detail. Consequently, 8 Hz should also not be enough to discriminate the fall features and, for this reason, the algorithm optimization and validation processes were repeated using the data at 100 Hz and undersampled to 50 Hz.

### 2.6. State Machine Thresholds Optimization

Since the main objective of this work is the creation of a robust fall detection algorithm independent of the on-body sensor location, samples from the three positions in dataset *DS-1* were mixed to train the algorithm.

The optimization process iterates over a set of thresholds of the state machine. For each threshold, an higher and lower bound of its possible value is defined. Then in each iteration, a random combination between each threshold values will be tested and the results will be summarized for all combinations. This optimization process was implemented following these steps:

1. Perform 10 times a random repeated stratified sampling of the dataset *DS-1* with train/test ratio of 0.7, meaning, 70% for the train set and 30% for the test set.
2. For each split, randomly undersample the majority class on the train set, in this case the non-fall movements, 10 times.
3. For each undersampled set, randomly generate 100 thresholds sets according to the allowed lower and higher bounds set for each parameter.
4. Train each set of thresholds with the corresponding train dataset. Save the 50 best and their respective test set.
5. Test the 50 selected sets of thresholds with the corresponding test set.
6. From the results obtained for all iterations of dataset splitting and undersampling, the 50 sets of thresholds that presented the best result during the test with the test part of the dataset were chosen.

The train/test ratio chosen was equal to 0.7 in order to take advantage of as much data as possible. Also, as it was also collected a validation set *DS-2*, the test part is only used for a preliminary choice and for this reason the majority of data can be used for training the algorithm.

#### 2.6.1. Sensitivity Level Sets Selection

After the iterative process explained in the last section, since it is not possible to obtain a perfect score in the detection of falls and non-falls, it was decided to choose three different sets of thresholds according to their levels of sensitivity: high, medium and low sensitivity sets of thresholds. With this objective, from the 50 results obtained in 6, the 10 sets of thresholds with the highest J-Index score were chosen in order to generate a Receiver Operating Characteristic (ROC) curve. To obtain the ROC, the value of sensitivity, true positive rate, obtained was plotted against the false positive rate (1-specificity). Then, the sets corresponding to the optimal point of the ROC curve, the point with highest specificity and the point with highest sensitivity were selected as the medium, low and high sensitivity sets of thresholds, respectively. This process was repeated using the results of the iterative process performed with the data at both 100 and 50 Hz of sampling rate.

### 2.7. Algorithm Validation

In order to validate the set of thresholds chosen after optimization at 100 and 50 Hz, they were tested using the dataset *DS-2*, Section 2.1. To analyze the results obtained on this test, they were firstly plotted in order to create a Total Operating Characteristic (TOC) curve [29]. In order to generate this curve, the hits, true positives (y axis), are plotted against the hits plus the false positives, this is, against the total of positive predictions (x axis) [29]. Therefore, this curve allows a better visualization of the balance between FAs and number of correctly detected falls for the six sets of thresholds (high, medium and low sensitivity levels for each frequency) chosen in the previous process Section 2.6.1. Since the fall detection algorithm evaluates each of the data samples (44.67 h of data at 100 Hz corresponds to more than 16 million samples), the number of non-fall cases that can possibly generate FAs is very large and difficult to represent. Thus, the maximum value of the x axis of the TOC chart, that represents the worst case in which all the non fall samples are wrongly classified as falls, is not represented. On the other hand, the maximum of the y axis, where the well classified falls are represented, is the amount of falls that the dataset *DS-2* contains. To analyze the TOC chart, it should be taken into account that when there were more points below the maximum of the y axis, the number of falls that were not detected increased. Also, if the algorithm has no FAs the number of well detected falls is the same as the number of well detected falls plus the false alarms, the value in x is equal to the value in y. Therefore, the more points there are to the right of the diagonal line, which represents the existence of no FAs, the higher the number of the FAs.

The performance of the fall detection algorithm when tested with the dataset *DS-2* is also analyzed calculating the sensitivity, precision and F-score. In order to better depict the prevalence of FAs and their possible impact on the daily life, besides the number of FAs and the precision, we also take into account the number of FAs per day, considering 16.5 h of daily usage [24].

#### Comparison of on Body Positions Performance

The validation results were also analyzed for each specific position where the wearable sensor can be used: chest, waist and pocket. Thus the dataset was divided by position where the sensor was placed during the acquisition. The amount of falls that the dataset *DS-2*, Section 2.1, contains are: 76 falls from both chest and waist and 120 falls from the pocket position. The pocket position is the most represented since in some acquisitions the subjects wore one sensor in each pocket. The objective is to compare the results obtained for the different positions and define the position and respective sampling rate for which the algorithm performs the best. With this objective in mind, the number of well detected falls, the sensitivity of the algorithm and the number of FAs were analyzed for each combination between sampling rate, sensitivity level and body position.

## 3. Results

### 3.1. Threshold Optimization—100 Hz

**Figure 1:**
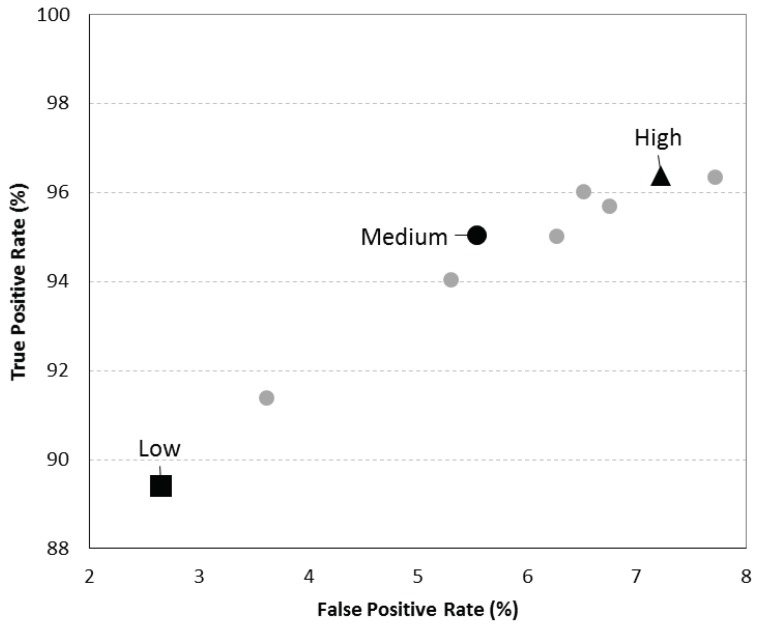
Receiver Operating Characteristic (ROC) curve with the 10 sets of thresholds that presented a better J-index when the algorithm was tested with the test set sampled at 100 Hz—Black square: Low sensitivity level; Black doth: Medium sensitivity level; Black triangle: High sensitivity level.

The ROC curve obtained after the threshold optimization process using the data at 100 Hz is presented in Figure 1. From this ROC curve the sets corresponding to the three levels of sensitivity, black points, were selected. The medium sensitivity point (black dot in Figure 1) presents 95.0% of sensitivity and 94.4% of specificity. The low sensitivity set which is also the one with higher specificity, black square, has 97.3% specificity and 89.4% of sensitivity, and the high sensitivity set (black triangle in Figure 1) has 96.4% of sensitivity and 92.8% of specificity.

### 3.2. Thresholds Optimization—50 Hz

**Figure 2:**
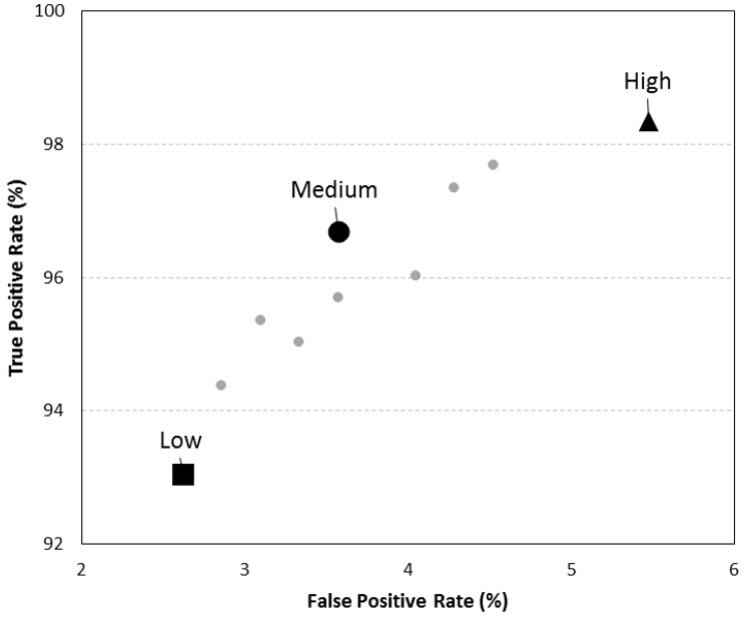
ROC curve with the 10 sets of thresholds that presented the best J-index when the algorithm was tested with the test set sampled at 50 Hz—Black square: Low sensitivity level; Black doth: Medium sensitivity level; Black triangle: High sensitivity level sensitivity.

The ROC curve with the 10 best results obtained in the test using the data undersampled at 50 Hz is presented in Figure 2. The black square in Figure 2 represents the results of the low sensitivity level, 97.4% of specificity and 93% of sensitivity. The medium sensitivity level is represented with the black dot, 96.4% of specificity and 96.7% of sensitivity. Lastly, the black triangle in Figure 2, represents the high sensitivity level, 98.3%, that had also 94.5% of specificity.

### 3.3. Comparison between 50 Hz vs. 100 Hz Sets

Table 2 summarizes the results for the three levels of sensitivity chosen in both 100 and 50 Hz optimization, already mentioned in Section 3.1 and Section 3.2. The results regard the test with 30% of the *DS-1*. As our iterative process randomly splits the *DS-1* in each iteration, the test results are obtained for different test sets. Therefore each set of thresholds was tested with a different test set. Even so, as can be observed in Table 2, the levels of sensitivity optimized for 50 Hz perform better than 100 Hz levels. The specificity increase was already expected when decreasing the sampling frequency, since with lower number of samples the possibility for outliers is reduced. This comparison between 50 and 100 Hz levels indicates that the algorithm performs better with data sampled at 50 Hz. However, since the test sets used are different, further validation with the dataset *DS-2* was conducted as described in Section 2.7.

### 3.4. Results by Type of Movement

Table 3 shows the results of the algorithm using the medium sensitivity level trained at 50 Hz set when tested with whole dataset *DS-1*. The results presented in this table can be generalized to the remaining sets of thresholds. As already referred in Section 2.1 the dataset *DS-1* includes some samples of dropping the sensor on a table because in some preliminary observations these movements were the main source of FAs. Even including these type of movements in the training set, as it can be observed in Table 3, these are the movements that more often generate FAs. When dropping the sensor quickly on the table the algorithm has accuracy of 54.8% while standing and 75% while seated. This prevalence of FAs exists due to the hard impacts of the sensor on the table which are similar to the impact of a person on the floor. Other movements that are often misclassified are the movements of standing or sitting, as are, for instance, falls with recovery or transition from a laying to a standing position.

Regarding fall classification there is no pattern for the most misclassified movements. The main factors that cause the misclassification of some samples are not the type of performed movements but the way each subject performs them. Depending on how comfortable the people is about falling to a mattress the way that each one falls is completely different. These differences are the major cause for the fall misclassifications of the algorithm.

### 3.5. Algorithm Validation in Continuous Usage

**Figure 3:**
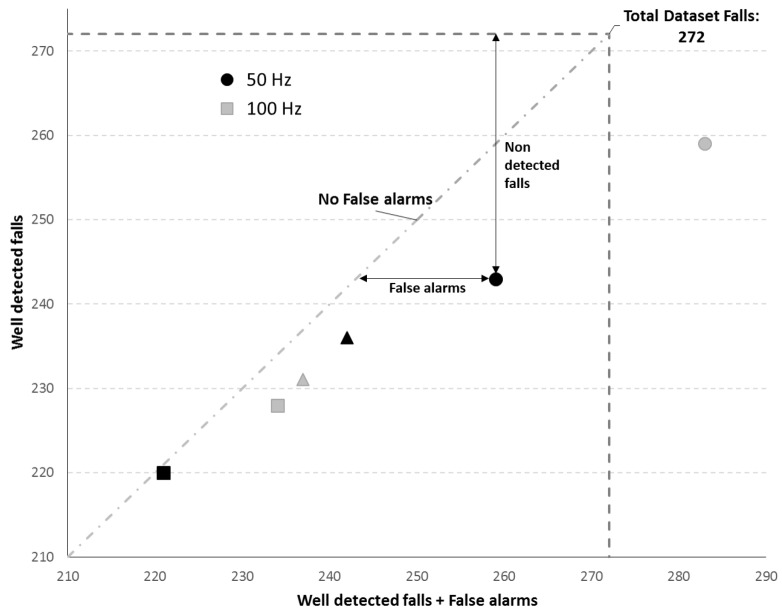
Total Operating Characteristic (TOC) curve with the results of the algorithm on *DS-2* using each set of thresholds chosen for each frequency. Legend: Squares—Low sensitivity levels; Triangles—Medium sensitivity levels; Circles—High sensitivity levels; Black marks—50 Hz; Grey marks—100 Hz.

The TOC curve obtained with the results from the validation test of the algorithm using each set of thresholds is presented in Figure 3, as explained in Section 2.7 of the methods. Analyzing this Figure 3 it can be observed, as expected, that results from the high sensitivity sets of thresholds, circles, are the ones closer to the “Total Dataset Falls” line which means that the number of falls correctly detected is, as expected, the highest for these sets. Simultaneously, these points are the ones that are further away to the right from the diagonal “No False alarms” line, which means that these sets are also the ones with higher number of FAs. The 50 Hz sets are, with exception of the medium sensitivity level, black triangle, closer to this line comparing with the 100 Hz sets. On other hand, the 100 Hz sets points, are always above the corresponding 50 Hz sets points, meaning that the number of well detected falls is higher. The exception to this behaviour is the medium sensitivity sets, since the 50 Hz set is able to detect a higher number of falls, as observed by the black triangle being above the grey triangle in Figure 3, while having the same number of FAs. In Section 3.3, the 50 Hz threshold sets showed the best performance regarding both sensitivity (fall detection rate) and specificity when tested in the test set of *DS-1*. It does not occur during this validation since the sensitivity is lower for the high and low sensitivity levels at 50 Hz when compared with the corresponding 100 Hz sets.

The remaining metrics obtained during this process of validation of the algorithm were summarized in Table 4. It shows that for all sets of thresholds, independently of the sampling rate, the sensitivity is always higher than 80%. As expected the set that has the lowest sensitivity, 80.9%, also has the lowest number of FAs. One FA was triggered using almost 45 h of data for the low sensitivity level thresholds set optimized at 50 Hz. Regarding the low sensitivity set at 100 Hz, the fall detection rate is higher, 84.9%. However, the number of FAs also increases from 1 to 6. These results are even more relevant when analysing the average of FAs per day, since the 100 Hz set would broadcast, in average, more than 2 FA per day while the 50 Hz set would only broadcast, in average, less than 0.5 FA per day.

On the other hand, the high sensitivity thresholds set optimized at 100 Hz is the one that presents the best fall detection rate, 95.2%. However, this set has also triggered a large amount of FAs (24) with an average of almost 9 FAs per day. Comparing the high sensitivity set optimized for 100 Hz, that presents 95.2% of sensitivity, with the one optimized for 50 Hz data, the sensitivity decreases to 89.3%. These values reveal that the amount of non detected falls increases from 13 to 29 between the 100 Hz and the 50 Hz sets. However, the precision increases from 91.5 to 93.8%, from the 100 to the 50 Hz set, revealing that using the data at 50 Hz can reduce the number of FAs in 3 FAs per day.

Regarding the medium sensitivity level, both 50 Hz and 100 Hz sets have performed similarly regarding the number of FAs, 6 at 100 and 5 at 50 Hz. However, the 50 Hz set has a higher value of fall detection rate, 86.8% vs. 83.8%. The number of FAs with these sets are lower than with the sets with high sensitivity level, however, they are still quite high with an average of 2.1 and 1.9 FAs per day, for the 100 Hz and 50 Hz sets, respectively.

Summing up, the use of a sampling rate of 50 Hz will benefit the algorithm’s performance by reducing the number of FAs, which is particularly relevant for the low sensitivity level mode.

#### 3.5.1. Comparison of on-Body Positions Performance

In Table 5, the results of the validation of the algorithm are compiled by the position of placement of the sensor. It becomes clear that the chest position is the position that presents higher performance regarding the number of FAs retrieved. Even at the highest sensitivity level it only retrieves 1 FA during 44 h. Wearing the sensor on the pocket significantly increases the probability of occurring an FA. For instance, using a high sensitivity set of thresholds the number of FA is 20 at 100 Hz and 12 at 50 Hz. Comparing the waist position with the remaining, we can observe that it presents a lower number of FAs than the pocket position but a higher number of false alarms than the chest position. These results were already expected since a higher variety of movements exists for both pocket and waist positions when compared to the chest, a more stable position during human routine movements.

Regarding the values of sensitivity, i.e., the fall detection rate, both pocket and waist outperform the position of the chest in the highest sensitivity level set. Using the sensor sampling at 100 Hz, on the pocket and set to the highest sensitivity this algorithm was able to detect 100% of falls. However, as already observed, with this set of thresholds the algorithm presents a higher number of FAs. Regarding the medium level sets of thresholds, the position that presents the best results is the waist when using the 50 Hz set, 89.5% of sensitivity. Using a low sensitivity level, the sensitivity values are higher for the pocket position at 100 Hz, 86.7%. At 50 Hz, sets of thresholds that had the highest sensitivity when using the low sensitivity setting were obtained using the sensor placed on the chest.

Comparing the whole possible combinations, the most reliable one is the algorithm with the medium sensitivity level, at 50 Hz, wearing the sensor on the user’s waist. For this position, and with this set of thresholds, the algorithm achieved 89.5% of sensitivity and did not have any FA.

As expected, some particular combinations of set of thresholds and positions present better performance than the overall results for all positions presented in Section 3.5. One of the main conclusions that can be retrieved from the analysis of both results is that the pocket is the position that influences the most the number of FA when using any of the high sensitivity set of thresholds. However, this position presents the highest values of sensitivity for these sets of thresholds which also increases the overall value, Table 4. Analysing of the results by position, we can conclude that, besides some exceptional results, for instance the number of FA retrieved when using the sensor on the pocket, the results can be generalized and the overall positions results reflect the expected performance of the algorithm for each position. Additionally, we can conclude that even training the algorithm with data from all three positions, the results obtained for a single position still have a performance comparable with some studies from literature. Therefore, the training methodology seems adequate in order to obtain an algorithm that is independent of the position.

## 4. Discussion

In this study we propose a fall detection algorithm that works independently of three different body locations, waist, chest and pocket. This algorithm is a low complexity accelerometer state machine that was implemented in a wearable device. The device is responsible for doing all the data processing and communicate the occurrence of a fall to, for example, a smartphone that would be responsible to transmit this information to a caregiver. In case the wearable contains a GSM feature, the fall alert could be directly broadcasted to the caregiver. Additionally, three different sensitivity/specificity optimization modes were developed, and a study about which accelerometer sampling rate would allow a better algorithm performance was carried on.

Different people have a different fall risk, depending on several intrinsic factors, such as, the physical activity level and specific health conditions. Factors like movements with huge impacts have similar patterns to falls which can influence the performance and reliability of the algorithm due to the probability of triggering false alarms. Therefore, it is particularly interesting to have an algorithm that allows an adjustment to the trade-off between specificity and sensitivity. This means that, if a person has a high risk of falling, a high sensitivity level of the algorithm should be used, while for a healthy person a low/medium sensitivity level should suffice. Thus, some sensitivity levels tested in this work should be considered for daily use. For instance, for a low risk patient, the best sensitivity level set at 50 Hz can be used, having 80.9% of fall detection rate and, in average, less than one FA per day, supporting three on-body positions. If the patient suffers from a condition that increases his fall risk, a high sensitivity level set should be selected, for instance, the 50 Hz set that, in our validation, had almost 90% of fall detection and 6 FA per day. The FA values will also depend on how active the user is, since active people will be more likely to have sudden movements that can be confused with falls and cause FAs. Since the data used in validation was acquired from young subjects, that are usually more active than elderly users, the results regarding the number of FAs can be biased representing a worst case scenario. For the subjects with high risk of falling, it is more important to have a better fall detection rate, due to the highest probability of occurring a fall. At the same time, these subjects are usually people with low mobility, that consequently have less movements that can generate FAs. Therefore one of the high sensitivity sets can be suitable for this group of high fall risk, since this sensitivity level has a high fall detection rate and the number of FAs would probably be reduced given the users’ lower mobility. The sets of thresholds deployed should also depend on the physical activity of the user. A high physically active person will have more movements that can have similar patterns to the falls and therefore sets with higher specificity should be deployed. A further validation with elders with different levels off physical activity and people with a high risk of falling is, for this reason, still required.

When varying the sampling rate of the accelerometer, the performance of the algorithm improves when the sampling rate decreases from 100 to 50 Hz, mainly regarding the FA rate. Gao et al. [30] have studied the performance of some single accelerometer activity classifiers for sampling rates between 10 and 200 Hz. They verified that the accuracy of the algorithm increases from 10 to 50 Hz and stabilizes above this frequency. These results are in accordance with those obtained in this study. Between the supported frequencies, 50 Hz was the frequency that showed the best results in our study considering the analysis of the trade-off between fall detection rate and FAs for the three sensitivity levels. The 100 Hz sets have shown, however, better performance regarding the fall detection rate, mainly for the high sensitivity set. Hence, the use of different sampling rates depending on the intended sensitivity level can be considered. For instance, when a high sensitivity level is chosen, the algorithm would use the data at 100 Hz and the respective high sensitivity sets of thresholds. For the low and medium levels the data would be undersampled to 50 Hz and the respective sets of thresholds, optimized to this sampling rate, would be used.

Table 6 shows the comparison between our work and some of the most important studies found in the literature. The features used for comparison are the type of the algorithm, the sampling rate of the used data, if the sensor can be placed in more than one body position (Adjustable Position column), if the algorithm was trained for more than one position in order to assess the best one (Multiple Position Train column) and if while using it, the levels of sensitivity or specificity of the algorithm can be adjusted (Adjustable Performance column). Additionally, some of the main results of the studies are presented. The main conclusions that can be drawn from the comparison are that our work can be distinguished from the remaining by the versatility of the positions where the wearable can be used and by the possibility of adjusting the algorithm’s performance according to the user’s needs. However, by comparing the “Results” column we can observe that some studies, mainly the ones that use Machine Learning or Kalman filter approaches, achieve better results than ours. In the remaining part of this discussion we present a more detailed comparison between the studies presented in this table and our work.

The validation of the algorithm performance by on-body position, presented in Section 3.5.1, have shown that among the three positions considered, the waist and the chest positions are the ones for which the algorithm achieved the best performance regarding the trade-off between FA and sensitivity. Some combinations like the medium sensitivity level set at 50 Hz on the user’s waist have shown a very balanced trade-off between sensitivity and FA, almost 90% of sensitivity and no FAs. The chest position has shown the best behaviour with respect to the trigger of FAs. The better performance of the algorithm on the chest and waist are in accordance with the results found in the studies of Kangas et al. [26] and Gjoreski et al. [27].

A low-power fall detector using accelerometer and barometer data was proposed by Wang et al. [23]. It was considered the usage of the sensor on the user’s chest. Their objective, as ours, is to develop an algorithm that runs on a wearable device and allows its battery to last as long as possible. They obtained a sensitivity of 93% and a FA rate of 0.023 alarms per hour, 0.3795 per day considering 16.5 h of daily usage. In our work, for instance, using the medium sensitivity level at 50 Hz wearing the sensor on the user’s chest, no FA were obtained. Additionally the value of fall detection rate is 8.8% lower than the obtained in [23]. In general the algorithm using the remaining set of thresholds outperforms their in terms of FA, but is outperformed regarding the obtained sensitivity. The worst performance of our algorithm using most of the sensitivity levels compared with their work could be explained, mainly, by the use of the barometer sensor in their work.

Pannurat et al. [18] developed a hybrid framework that combines activity classification using machine learning and a rule-based knowledge representation for detection of different phases of falls. Similarly to our algorithm, it also works for several positions, namely, head, arm, wrist, ankle, chest, side waist, front waist and thigh. For all the positions, the specificity of their algorithm is lower than the one obtained with our algorithm using any sensitivity level set. When testing their best algorithm with a dataset containing activities of daily living they found false alarm rates below 0.05% for both waist and chest positions. The dataset they used has smaller periods of data of each activity (15 s) when compared to our continuous dataset Section 2.1. As they use 0.5 s windows, for the chest position they have 61,200 data samples. Therefore, the value of false alarm rate reported, 0.05%, represent a total of 6 FAs in less than half an hour of data. With some specific levels of thresholds for both considered positions, chest and waist, our algorithm did not trigger any false positive in almost 45 h of data. The fall detection rate is similar on both algorithms. Although our algorithm presents better results, a further comparison using the same dataset for validation is still required. Even so, the algorithm presented here has some advantages when compared with the one presented by Pannurat et al. [18] such as the independence of the on-body sensor position for the three positions considered (waist, chest and pocket), as well as the fact that it does not require a calibration step before changing the wearable position.

Nowadays, more sophisticated algorithms using machine learning or complex processing algorithms like a Kalman filter, have shown high rates of accuracy, nearly 100% [19,20,25]. However, the MCU used in this work does not support floating point calculations neither hardware divisions, therefore, more complex operations like trigonometric calculations, square roots or divisions require approximations that can affect the algorithm accuracy and affect the algorithm execution speed and battery performance. Moreover, the use of such complex operations would increase the firmware size, which compromise an already scarce resource—memory. From the 16 kb of RAM of the device, only approximately 3 kb were available to implement our algorithm. With this amount of RAM, even disregarding the use of complex calculations, it is not possible to implement any windowing technique since, for instance, 2 windows of 3 s with 4 channels of accelerometer data (x, y, z and timestamp), with a precision of only 16 bits, require 5 kB of memory. Therefore, due to these limitations, it is not possible to implement more complex algorithms such as machine learning algorithms.

A study of Bagatá et al. [31] demonstrated that most common algorithms decrease their accuracy when tested with real falls instead of simulated ones. For this reason, the algorithm presented in this work still requires validation with real falls. Even though this data is really difficult to obtain since falls are rare and unpredictable events [12].

## 5. Conclusions

In this work we present a fall detection algorithm implemented in a wearable device that can be used in three different body positions, chest, waist and pocket. It uses single accelerometer data and classifies the movements using a state machine. Even while having to respect hardware constraints that require a very simple algorithm and impose implementation approximations, and being optimized and tested for three different on-body positions, it presents a performance level similar to more complex or single position algorithms currently presented in the literature. Considering the single position performance validation, the algorithm shows also comparable results with the ones presented in the literature. Additionally, it shows some versatility since it can be adjusted to three different levels of sensitivity that can be used to better suit the subjects’ needs depending on different risks of falling and mobility patterns. In this study it has also been showed that decreasing the accelerometer sampling rate does not largely affect the accuracy of the detection, being even beneficial for avoiding false alarms. The decrease in the accelerometer sampling rate has a positive side effect in decreasing the demand for the processing capabilities, resulting in a more suitable use of the algorithm in wearable devices.

To summarize, the fall detection algorithm described in this work presents a reliable, simple, and wearable solution for automatic fall detection. This is a versatile solution that can be adapted to different groups of people with different fall risk levels. Also, it can be used in different on-body positions, without requiring any calibration step, which makes this system less intrusive and easier to use. 

## Figures and Tables

**Table 1:** Description of movements present on dataset *DS-1*.

Non-Fall Movements	Samples
While seated slowly drop sensor on the table	99
While seated quickly drop sensor on the table	68
While standing quickly drop sensor on the table	73
While standing slowly drop sensor on the table	82
Stumble	67
Sit on a lower chair, wait 10 s and get up	63
Left lateral fall with recovery	67
Walking and sitting on a chair	65
Lay, wait 10 s, stand, wait 10 s	70
Forward Fall with recovery	67
Backward fall with recovery	67
Catch an object from the floor while walking	67
Walk a few meters	68
Cough and sneeze	67
Sit, wait 10 s, stand, wait 10 s	70
Run a few meters	67
Right lateral fall with recovery	66
While seated slightly lift the body	31
Sitting on a chair	34
Laying on a bed	31
Bend and pick an object from the floor	18
Get up from laying and stand	59
Get up from laying and sit	30
Walk	3
**Total**	1399
**Fall Movements**	
Sited on a chair, stand up, walk and fall	64
Backward fall ending lying	70
Forward fall with rotation, ending in lateral left position	67
Backward fall ending sitting	67
Forward fall with rotation, ending in lateral right position	67
Walk few meters and then right lateral fall	67
Lateral fall to the left ending lying flat	67
Lateral fall to the right ending lying flat	67
Backward fall ending in lateral position	66
Walk few meters and fall backwards	66
Walk few meters and left lateral falls	67
Forward fall on the knees	70
Forward fall ending lying flat	67
Forward fall with forward arm protection	70
Walk few meters and fall forward	67
**Total**	1009

**Table 2:** Comparison of 100 Hz and 50 Hz sets of thresholds when tested with each respective test set, 30% of the dataset *DS-1*.

Sensitivity	High		Medium		Low	
Frequency (Hz)	100	50	100	50	100	50
Sensitivity (%)	96.4	98.3	95.0	96.7	89.4	93.0
Specificity (%)	92.8	94.5	94.4	96.4	97.3	97.4
J index (%)	89.2	92.8	89.4	93.1	86.7	90.4

**Table 3:** Results of the medium sensitivity level of thresholds trained with data at 50 Hz with whole dataset *DS-1*. The results are divided by type of movement in order to analyze the movements that are more often misclassified.

Non-Fall Movements	Samples	Pred Fall	Pred Non Fall	Acc (%)
While seated slowly drop sensor on the table	99	5	94	94.9
While seated quickly drop sensor on the table	68	17	51	75.0
While standing quickly drop sensor on the table	73	33	40	54.8
While standing slowly drop sensor on the table	82	1	81	98.8
Stumble	67	0	67	100
Sit on a lower chair, wait 10 s and get up	63	0	63	100
Left lateral fall with recovery	67	1	66	98.5
Walking and sitting on a chair	65	0	65	100
Lay, wait 10 s, stand, wait 10 s	70	2	68	97.1
Forward Fall with recovery	67	0	67	100
Backward fall with recovery	67	0	67	100
Catch an object from the floor while walking	67	0	67	100
Walk a few meters	68	0	67	100
Cough and sneeze	67	0	67	100
Sit, wait 10 s, stand, wait 10 s	70	0	70	100
Run a few meters	67	1	66	98.5
Right lateral fall with recovery	66	0	66	100
While seated slightly lift the body	31	0	31	100
Sitting on a chair	34	0	34	100
Laying on a bed	31	0	31	100
Bend and pick an object from the floor	18	0	18	100
Get up from laying and stand	59	0	59	100
Get up from laying and sit	30	0	30	100
Walk	3	0	3	100
**Total**	1399	60	1339	95.7
**Fall Movements**				
Sited on a chair, stand up, walk and fall	64	56	8	87.5
Backward fall ending lying	70	70	0	100
Forward fall with rotation, ending in lateral left position	67	65	2	97.0
Backward fall ending sitting	67	61	6	91.0
Forward fall with rotation, ending in lateral right position	67	63	4	94.0
Walk few meters and then right lateral fall	67	67	0	100
Lateral fall to the left ending lying flat	67	65	2	97.0
Lateral fall to the right ending lying flat	67	64	3	95.6
Backward fall ending in lateral position	66	66	0	100
Walk few meters and fall backwards	66	66	0	100
Walk few meters and left lateral falls	67	62	5	92.5
Forward fall on the knees	70	70	0	100
Forward fall ending lying flat	67	62	5	95.5
Forward fall with forward arm protection	70	70	0	100
Walk few meters and fall forward	67	63	4	94.0
**Total**	1009	970	39	96.1

**Table 4:** Results of the test using the *DS-2* with both 50 and 100 Hz sets of thresholds.

Sensitivity Levels	High		Medium		Low	
Amount of data	44.67 h					
Number of falls	272					
$S_{rate}$ (Hz)	100	50	100	50	100	50
True Positives	259	243	228	236	231	220
False Alarms	24	16	6	5	6	1
Sensitivity %	95.2	89.3	83.8	85.6	84.9	80.9
Precision %	91.5	93.8	97.5	97.5	97.5	99.5
F-score %	93.3	91.5	90.1	91.8	90.8	89.3
FA per day	8.9	5.9	2.1	1.9	2.1	0.4

**Table 5:** Results by position and by frequency obtained by our algorithm when tested using the dataset *DS-2*.

Sensitivity Level		High		Medium		Low	
$S_{rate}$ (Hz)		100	50	100	50	100	50
**Chest**	TP	67	66	68	64	63	63
	Sens (%)	88.2	86.8	89.4	84.2	82.9	82.9
	FA	1	1	1	0	0	0
**Pocket**	TP	120	108	98	101	104	96
	Sens (%)	100.0	90.0	81.7	84.2	86.7	80.0
	FA	20	12	0	5	6	0
**Waist**	TP	72	69	62	68	64	61
	Sens (%)	94.7	90.8	81.6	89.5	84.2	80.3
	FA	3	3	5	0	0	1

**Table 6:** Comparison between the developed work and some of the most important studies found in the literature. Legend—SM—State Machine; TB—Threshold Based; ML—Machine Learning; RB—Rule Based; Sens—Sensitivity; Spec—Specificity; Acc—Accuracy; #FA—Number False Alarms.

Work	Algorithm	$S_{\mathrm{rate}}$ (Hz)	Adjustable Position/Multiple Position Train	Adjustable Performance	Results (%)
Our work	SM	100 or 50	Yes/Yes	Yes	Sens 96.7 Spec 96.4
Kangas et al. 2008 [26]	TB	50	No/Yes	No	Sens 97.0 Spec 100.0
Gjoreski et al. 2011 [27]	ML + TB	6	No/Yes	No	Acc 71.0 #FA 5
Wang et al. 2016 [23]	TB	6 or 50	No/No	No	Sens 93 Spec 87
Pannurat et al. 2017 [18]	RB + ADL Classification	15	Yes/Yes	No	Sens 93.6 Spec 88.3
Sucerquia et al. 2018 [19]	Kalman filter + TB	25	No/No	No	Sens 99.3 Spec 99.4
Özdemir et al. 2014 [20]	ML	25	No/Yes	No	Sens 100.0 Spec 99.8
Özdemir 2016 [25]	ML	25	No/Yes	No	Acc 99.9

## Abbreviations

The following abbreviations are used in this manuscript:

ANN	Artificial Neural Networks
CPU	Central Processing Unit
FA	False Alarm
FN	False Negative
FP	False Positive
$J_{index}$	Youden Index
ROC	Receiver Operating Characteristic
Sens	Sensitivity
Spec	Specificity
$S_{rate}$	Sampling rate
TOC	Total Operating Characteristic
TN	True Negative
TP	True Positive
